# Novel and Future Therapeutic Drugs for Advanced Mycosis Fungoides and Sézary Syndrome

**DOI:** 10.3389/fmed.2019.00116

**Published:** 2019-05-29

**Authors:** Tomonori Oka, Tomomitsu Miyagaki

**Affiliations:** Department of Dermatology, Graduate School of Medicine, The University of Tokyo, Tokyo, Japan

**Keywords:** mycosis fungoides, Sézary syndrome, peripheral T-cell lymphoma, clinical trial, novel therapeutic agents

## Abstract

Mycosis fungoides (MF) and Sézary syndrome (SS) are the most common subtypes of cutaneous T-cell lymphoma. The majority of MF cases present with only patches and plaques and the lesions are usually limited to the skin. On the other hand, in some cases, patients show skin tumors or erythroderma followed by lymph node involvement and rarely visceral organ involvement. SS is a rare, aggressive cutaneous T-cell lymphoma marked by exfoliative erythroderma, lymphadenopathy, and leukemic blood involvement. Because patients with relapsed or refractory MF/SS display a poor prognosis and the current treatment options are characterized by high rates of relapse, there is unmet need for the efficient treatment. This review provides a discussion of the recent and future promising therapeutic approaches in the management of advanced MF/SS. These include mogamulizumab, brentuximab vedotin, alemtuzumab, immune checkpoint inhibitors, IPH4102 (anti-KIR3DL2 antibody), histone deacetylase inhibitors (vorinostat, romidepsin, panobinostat, belinostat, and resminostat), pralatrexate, forodesine, denileukin diftitox, duvelisib, lenalidomide, and everolimus.

## Introduction

Cutaneous T-cell lymphoma (CTCL) comprises a clinically/pathologically heterogeneous group of uncommon non-Hodgkin's lymphomas that manifest primarily in the skin. Mycosis fungoides (MF) is the most common CTCL subtype that accounts for around 60% of CTCL (1). MF is generally an indolent lymphoma with slow progression over years or even decades. Typically, the initial lesions in MF are flat and erythematous skin patches, which evolve over a variable period of time into palpable plaques characterized by well-demarcated edges. In limited cases, plaques can be followed by tumors and those patients have patch, plaque, and tumor lesions simultaneously on different parts of the body. In some cases, skin lesions develop into erythroderma similar to Sézary syndrome (SS). In MF cases with tumors or erythroderma (advanced MF), lymph node or visceral involvement is sometimes observed and such cases present a poor prognosis. SS is a much rarer variant, accounting for only 3% of CTCL (1). Characteristics of SS are generalized erythroderma (defined as affecting > 80% of total body surface area), lymphadenopathy, and presence of circulating tumor cells in the blood. Progression of SS is usually more rapid compared to that of MF.

Although MF and SS are classified as distinct, separate entities, the same clinical staging system and therapeutic approaches have been used (1, 2). Patients with MF having limited T1 stage (limited patches, papules, and/or plaques covering < 10% of the skin surface) have a similar life expectancy to that of control populations (3). In addition, patients with early stage MF (stage I and IIA) have a good prognosis (a median survival: 15.8 years or more), while patients with advanced stage MF/SS (stage IIB or more) have a poor prognosis (a median survival: 4.7 years or less) (3). Current treatment consists of skin-directed therapies, such as topical corticosteroid, topical mechlorethamine, topical bexarotene, ultraviolet phototherapy, total skin electron beam therapy, and localized radiotherapy (2), for early stage disease and systemic therapies for advanced stage. For early stage MF confined to the skin, therapeutic concept is to control symptoms by skin-directed therapies with the lowest possible therapy-related side effects, as durable remissions cannot be achieved by early aggressive chemotherapy (4). For advanced stages of MF and SS, there is a variety of systemic therapies available, some of which are used from decades ago and some recently. However, currently available drug therapies are not curative treatment and the only option for curing MF/SS is stem cell transplantation (5).

As MS/SS have the chronic and recurrent nature, repeated treatment courses and maintenance regimens are necessary for disease control. Although there are available active systemic therapeutic strategies, including cytotoxic chemotherapy and biological therapy, better treatments of advanced stage and refractory MF/SS are desired by both patients and physicians. Purpose of the present paper is to review the clinical results obtained in clinical trials of novel currently used and future promising therapies for advanced MF/SS patients (Table 1).

**Table 1:** Summary of the results of clinical trials of single-agents in cutaneous T-cell lymphoma or peripheral T-cell lymphoma including a given number of mycosis fungoides or Sézary syndrome patients.

	**Ref**	**Phase**	**Subtypes^†^**	**Number of patients^‡^**	**ORR, %**	**CRR, %**	**Median DOR**	**PFS**	**Approval year^§^**		
									**FDA**	**EMA**	**PMDA (Japan)**
Mogamulizumab	(6)	2%	MF/pcALCL	8(7)	37.5	0%	ND	ND	2018%	2018%	2014%
	(7)	1/2	MF/SS	38%	36.8	7.9	10.4 months	50% at 11.4 months			
	(8)	3%	MF/SS	186%	28%	3%	14.1 months	50% at 7.7 months			
Brentuximab vedotin	(9)	2%	MF/SS	30%	70%	3%	ND	54% at 12 months	2017%	2017%	-
	(10)	3%	CD30^+^ MF	28%	54%	7%	8 months	ND			
	(11)	3%	CD30^+^ MF	48%	65%	10%	15.1 months	50% at 16.7 months			
Alemtuzumab	(12)	2%	MF/SS	22%	55%	32%	ND	ND	-	-	-
Nivolumab	(13)	2%	MF	Ongoing					-	-	-
Pembrolizumab	(14)	2%	MF/SS	Ongoing					-	-	-
IPH4102	(15)	1%	MF/SS	Ongoing					-	-	-
	(16)	1%	SS	Ongoing							
Vorinostat	(17)	2%	MF/SS	74%	29.7	0%	6 months or more	ND	2007%	2004 (orphan), 2009 withdrawn	2011%
	(18)	2%	MF/SS	33%	24.2	0%	3.8 months	50% at 3 months			
Romidepsin	(19)	2%	MF/SS	71%	33%	7%	13.7 months	ND	2009%	2005 (orphan), 2012 refused (PTCL)	2018 (PTCL)
	(20)	2%	MF/SS	96%	34%	6%	15 months	ND			
Panobinostat	(21)	2%	MF/SS	139%	17.3	1.4	ND	ND	-	-	-
Belinostat	(22)	2%	MF/SS/other CTCL^#^	29 (23)	13.8	10.3	3 months	ND	2014 (PTCL)	2012 (orphan, PTCL)	-
Pralatrexate	(24)	2%	MF	109%	58%	16.7	4.4 months	50% at 5.3 months	2009 (PTCL)	2007 (orphan), 2012 refused (PTCL)	2018 (PTCL)
Forodesine	(25)	2%	MF/SS	144%	16%	1%	8.7 months	ND	-	2007 (orphan), 2012 refused (PTCL)	2018 (PTCL)
Denileukin diftitox	(26)	3%	MF/SS/other CTCL^#^	100 (91)	44%	10%	7.8 months	50% at 26.5 months	1999%	2001%	-
Duvelisib	(27)	1%	MF/SS/pcALCL	19 (9)	31.6	0%	ND	50% at 4.5 months	-	-	-
Lenalidomide	(28)	2%	MF/SS	Ongoing					-	-	-
Everolimus	(29)	2%	MF	Ongoing					-	-	-

† *When data regarding patients with MF/SS is separable in the original paper, data on MF/SS patients is shown. When inseparable, data on CTCL patients is shown*.

‡ *When data regarding patients with MF/SS is inseparable in the original paper, the number of patients with MF/SS is shown in parentheses*.

§ *When the drug was approved or refused not for CTCL but for PTCL, the comment “(PTCL)” is added. When the drug was approved as orphan drug from EMA, the comment “(orphan)” is added*.

# *Other CTCL includes pcALCL, peripheral T-cell lymphoma, not otherwise specified, and subcutaneous panniculitis-like T-cell lymphoma*.

*Ref, reference; ORR, overall response rate; CRR, complete response rate; DOR, duration of response; PFS, progression free survival; FDA, food and drug administration; EMA, European medicines agency; PMDA, pharmaceuticals and medical devices agency; MF, mycosis fungoides; pcALCL, primary cutaneous anaplastic large cell lymphoma; ND, not described; SS, Sézary syndrome; PTCL, peripheral T-cell lymphoma; CTCL, cutaneous T-cell lymphoma*.

## Mogamulizumab

C-C chemokine receptor 4 (CCR4) is the receptor for thymus and activation-regulated chemokine and macrophage-derived chemokine and is involved in skin trafficking of type 2 helper T cells and regulatory T cells. CCR4 is also consistently expressed on the surface of tumor cells in T-cell malignancies, such as CTCL, including MF and SS, adult T-cell leukemia-lymphoma, and peripheral T-cell lymphoma (PTCL) (30–33). Mogamulizumab is a humanized IgG1 κ monoclonal antibody with a defucosylated Fc region, which selectively binds to CCR4. The antibody exerts its antitumor activity by antibody-dependent cellular cytotoxicity (34). First, mogamulizumab has been approved in Japan for relapsed or refractory CCR4^+^ adult T-cell leukemia-lymphoma (2012), PTCL (2014), and CTCL (2014) (35).

Before the approval of mogamulizumab in Japan, seven patients with MF had been enrolled in a multicenter phase 2 study for patients with relapsed PTCL and CTCL in Japan (6). Intravenous infusions of 1.0 mg/kg mogamulizumab were administered to patients once per week for 8 weeks. The overall response rate (ORR) for MF patients was 28.6% [all partial response (PR) with no complete response (CR)]. A phase 1/2 study was also conducted for 38 patients with pretreated CTCL (MF and SS) in USA. Mogamulizumab was administered once weekly for 4 weeks using an escalation scheme (0.1 mg/kg and subsequent doses of 0.3 and 1.0 mg/kg) followed by 1.0 mg/kg every 2 weeks until disease progression or withdrawal. The ORR was 36.8% (CR 7.9% and PR 28.9%). Mogamulizumab was more effective for patients with SS than those with MF; ORR was 47.1% in SS (*n* = 17) and 28.6% in MF (*n* = 21). Eighteen of 19 (94.7%) patients with blood involvement had a response in blood, including 11 CRs (7). In an international, open-label, randomized, controlled phase 3 trial in patients with relapsed or refractory MF/SS (MAVORIC study), mogamulizumab (1.0 mg/kg once weekly for 4 weeks followed by every 2 weeks) significantly showed the high ORR and prolonged progression free survival (PFS) compared with 400 mg/day vorinostat (8). The ORR of mogamulizumab was 28% (21% in MF and 37% in SS), while the ORR of vorinostat was 4% (8). The median PFS was 7.7 months for the mogamulizumab group, compared with 3.1 months for vorinostat. Compartment response rates were 78/186 (42%) in skin, 83/122 (68%) in blood, 21/124 (17%) in lymph nodes, and 0/3 (0%) in viscera, suggesting that mogamulizumab is effective especially for blood involvement. In all studies, mogamulizumab showed an acceptable safety profile and common toxicities included nausea, chills, headache, fever, diarrhea, pruritus, and infusion reactions. Based on these results, mogamulizumab was approved for the treatment of patients with CTCL who have received at least 1 prior systemic therapy by the US Food and Drug Administration (FDA) and European Medicines Agency (EMA) in 2018.

## Brentuximab Vedotin

CD30 is a cell membrane protein that belongs to the tumor necrosis factor receptor family. CD30 was originally discovered on Reed-Sternberg cells of Hodgkin's lymphoma, and its expression was subsequently demonstrated on subsets of non-Hodgkin lymphoproliferative disorders, notably systemic, and primary cutaneous anaplastic large T-cell lymphoma (ALCL) and lymphomatoid papulosis. CD30 is also expressed on tumor cells of some MF/SS cases at various levels, and cases with large cell transformation frequently show higher expression. Brentuximab vedotin (BV) is an antibody-drug conjugate composed of the cytotoxic antitubulin agent monomethyl auristatin E (MMAE) and a chimeric monoclonal anti-CD30 antibody (36). After BV binds to CD30, the antibody-drug conjugate is internalized, and the antibody is cleaved by the lysosome, leading to the intracellular release of MMAE (37). MMAE inhibits tubulin polymerization and consequently disrupts the microtubule network within the cells causing cell cycle arrest and apoptosis. In addition, a small fraction of MMAE is released from CD30^+^ cells, killing neighboring cells in the tumor microenvironment in a CD30-independent manner (36, 37). BV has received regulatory approval in more than 65 countries for the treatment of relapsed or refractory Hodgkin's lymphoma and systemic ALCL (38).

The results of two phase 2 studies of BV for CD30^+^ CTCL including MF/SS were reported in 2015. In one phase 2 trial of 30 evaluable patients with pretreated CD30^+^ MF/SS by Kim et al, the patients received up to 16 cycles of BV (1.8 mg/kg) every 3 weeks. The ORR was observed in 21 (70%) of 30 patients (CR in one patient and PR in 20 patients), and patients with CD30 expression <5% exhibited a decreased probability of response compared with patients with CD30 expression >5%. (9). In the other trial of BV for 48 pretreated patients with primary cutaneous CD30^+^ lymphoproliferative disorders, 28 patients with CD30^+^ MF were included (10). BV was administered intravenously at 1.8 mg/kg every 3 weeks for a maximum of eight doses. The ORR in MF patients was 54% with CR in two cases and the response was independent of CD30 expression. Based on these promising results, the international randomized phase 3 trial (ALCANZA study) for pretreated CD30^+^ CTCL (MF or primary cutaneous ALCL) had been conducted recently to compare BV against the chosen standard therapy by physicians (methotrexate or bexarotene). In this clinical trial, included cases expressed the CD30 molecule on at least 10% of the skin infiltrate BV (1.8 mg/kg every 3 weeks) and methotrexate (5–50 mg weekly) or bexarotene (300 mg/m^2^ daily) were administered until disease progression or the development of major toxicity. Among the enrolled patients, 97 patients with MF were included. Forty-eight patients were treated with BV and the remaining 49 patients were treated with methotrexate or bexarotene. The ORR lasting at least 4 months was increased in the BV cohort compared with the physician's choice cohort (50 vs. 10%). Five patients achieved CR with BV, while methotrexate or bexarotene failed to achieve CR in any patient. After a median follow-up time of 17.5 months, the median PFS was 15.9 months for patients in the BV cohort and 3.5 months for patients in the methotrexate or bexarotene cohort (11). Peripheral neuropathy was the most frequent adverse event (AE) and was observed in 67% of patients undergoing treatment with BV. After a median 22.9 months of follow-up, 82% of patients with peripheral neuropathy experienced improvement or resolution. Other common side effects reported during the study included nausea, diarrhea, vomiting, alopecia, itching, fever, and loss of appetite. These data suggested that BV can be a preferable treatment option for the treatment of MF when biopsy samples have 10% or more CD30^+^ malignant cells. In 2017, FDA and EMA approved BV for the treatment of adult patients with CD30^+^ MF who have received prior systemic therapy.

## Alemtuzumab

CD52 is a small glycopeptide composed of 12 aminoacids expressed on the cell surface of several different types of leukocytes, including normal and malignant T lymphocytes. Alemtuzumab is a humanized IgG1 antibody that targets the CD52 antigen. The phase 2 study of alemtuzumab in patients with advanced MF/SS who did not respond adequately to treatment with at least PUVA, radiotherapy, or chemotherapy, showed that the ORR was 55% with 32% CR and 23% PR (12). The effect was better on erythrodermic patients (69% ORR with 38% CR) than on patients with plaques or tumors (40% ORR with 30% CR). In that study, alemtuzumab was administered using escalating doses (5, 10, 30 mg intravenously on days 1–3) and then 30 mg/day three times a week for up to 12 weeks. Because AEs of alemtuzumab such as infusion reaction, hematologic toxicity, and infectious complications were severe, clinical trials of low-dose alemtuzumab were performed for CTCL. In 14 patients with SS treated with subcutaneous low-dose alemtuzumab (3 mg on day 1, 10 mg on day 3, then 15 mg on alternating days or 3 mg on day 1, then 10 mg on alternating days), the ORR was 85.7% with 21.4% CR and 64.3% PR (39). Infectious episodes were observed only in patients treated with 15 mg alemtuzumab. These studies suggest that low-dose alemtuzumab can be an effective treatment for erythrodermic MF/SS with acceptable safety. Consistently, a recent report on 23 patients with leukemic involvement treated with low-dose alemtuzumab (10 mg subcutaneously, three times a week) described that 13 of 17 patients presented with erythroderma showed CR and that the remaining 4 patients could be controlled by following skin-directed therapy alone. In contrast, CR was not achieved in any patient with discrete patches, plaques, or tumors (40).

## Immune Checkpoint Inhibitors

Immune checkpoint molecules, such as cytotoxic T lymphocyte-associated protein 4 (CTLA-4) and programmed cell death protein 1 (PD-1), act as negative regulators that inhibit normal T-cell responses to avoid the emergence of pathological self-reactivity. On the other hand, cancers occasionally have the capacity to avoid anti-tumor immunity by abusing such immune checkpoint molecules. Thus, immune checkpoint inhibitors can antagonize the immunosuppressive interaction between the tumor cells and T cells and improve antitumor immune T-cell responses. In recent years, the efficacy of immune checkpoint inhibitors blocking the CTLA-4 and PD-1 pathways has been confirmed by several clinical trials in a variety of cancers. PD-1-blocking antibodies (nivolumab and pembrolizumab) and CTLA-4-blocking antibody (ipilimumab) achieved durable objective responses and improved OS in patients with solid tumors (23, 41–44) and hematologic malignancies, including Hodgkin's lymphoma (45). Concerning hematological malignancies, in 2016, nivolumab was approved for the treatment of patients with classical Hodgkin lymphoma that has relapsed or progressed after autologous hematopoietic stem cell transplantation and the following post-transplantation BV by FDA. Subsequently, FDA approved pembrolizumab for the treatment of refractory primary mediastinal large B-cell lymphoma patients in 2018.

Current data suggest that the PD-1, PD-L1/PD-L2 pathway may play a significant role in preventing immune-driven eradication of MF/SS tumor cells. Expression of PD-1 and PD-L1 has been detected in tumor cells of various morphological subsets of MF (46) as well as tumor cells circulating in the peripheral blood of SS (47). A recent phase 1b study of nivolumab in 81 patients with relapsed or refractory hematologic malignancy included 13 patients with MF. The ORR in MF patients was 15% (all PR) with 59% stable disease (SD) and the median PFS was 10 weeks (13). Khodadoust et al. presented preliminary data from a multicenter phase 2 open label study of pembrolizumab in 24 advanced and refractory CTCL patients (9 MF, 15 SS) (14). The ORR was 37.5% with 1 CR, 8 PR, and 9 SD, and the median PFS has not yet been reached. Of the 9 responding patients, 6 patients had 90% or greater decrease in modified Severity Weighted Assessment Tool score. Treatment was well-tolerated with a toxicity profile which was consistent with prior studies (48), although a notable skin flare reaction was developed in 40% of SS patients. Although it is necessary to wait for the results of several ongoing clinical trials using immune checkpoint inhibitors such as nivolumab, ipilimumab, and durvalumab (anti-PD-L1 antibody), immune checkpoint inhibition can be a novel strategy to treat advanced MF/SS.

## IPH4102 (anti-KIR3DL2 Antibody)

KIR3DL2 (CD158k), a member of the highly polymorphic killer-cell immunoglobulin-like receptor family, has the capacity to bind to MHC class I and transduce an inhibitory signal. KIR3DL2 is expressed on subsets of normal CD8^+^ T cells and NK cells, but not on normal CD4^+^ cells (49). On the other hand, several studies demonstrated that KIR3DL is expressed by neoplastic CD4^+^ T cells in SS, advanced MF, and primary cutaneous ALCL (50–54). The relative specific expression of KIR3DL2 on the malignant CTCL cells makes it an ideal therapeutic target. IPH4102 is a humanized, monoclonal antibody specific toward KIR3DL2 which lacks cross-reactivity with other members of the human killer-cell immunoglobulin-like receptor family. IPH4102 selectively and efficiently can deplete KIR3DL2^+^ cells including primary Sézary cells through antibody-dependent cell cytotoxicity and phagocytosis (55).

Preliminary results from the phase 1 study were presented at the 2017 European Organization for Research and Treatment of Cancer: Cutaneous Lymphoma Task Force in London (15). The aim of the trial is to characterize IPH4102 safety profile and identify the maximum tolerated dose and recommended phase 2 dose. A total of 25 patients, including 20 patients with SS, four patients with MF, and one patient with CD4^+^ CTCL (neither MF nor SS), have been treated at the 10 preplanned ascending dose levels (0.0001–10 mg/kg). All patients had relapsed after or had been refractory to at least two prior systemic therapies. The ORR was 44% (1 CR and 10 PR). Two patients achieved a near CR (>90% reduction in skin involvement). The median duration of response (DOR) was 8.2 months, and the median PFS was 9.8 months. As IPH4102 was safe and well-tolerated in those dose-escalation cohorts, expansion cohorts started at the flat dose of 750 mg in 2017. Preliminary results of expansion cohorts were presented at the 60th American Society of Hematology annual meeting in 2018 (16). The study included 35 SS patients with at least two prior systemic therapies. The ORR was 42.9% (5.7% CR and 37.2% PR) with a favorable safety profile. The median DOR was 13.8 months and the median PFS was 11.7 months. Preliminary phase 1 data suggest that IPH4102 is both efficacious and well-tolerated. A global, multi-cohort, phase 2 study evaluating the potential of IPH4102 in different subtypes of T-cell lymphoma will be initiated this year (NCT03902184).

## HDAC Inhibitors

Histone deacetylase (HDAC) inhibitors have the capacity to increase acetylation of histones and other proteins, which exerts chromatin remodeling, promotion of tumor suppressor gene transcription, and apoptosis, resulting in antitumor activity. Its clinical activity is largely confined to hematologic malignancies, particularly CTCL (56). HDAC inhibitors have the prevalent AEs of fatigue, thrombocytopenia, diarrhea, and nausea in common (57).

Although vorinostat is not a novel drug, we referred to the drug in this paragraph, because it is the first approved HDAC inhibitor. Vorinostat is an oral competitive inhibitor of class I/II HDAC enzymes. In the pivotal phase 2B multicenter trial, 400 mg of vorinostat was administered daily to 74 stage IB-IVA MF/SS patients, who were previously treated with two or more prior systemic therapies, until disease progression or intolerable toxicity (17). The ORR was 29.7% (22/74) and all initial responses were confirmed PR. The other phase 2 clinical trial showed similar results (18). Eight of 33 patients (24.2%) with refractory MF/SS who had received a median of 5 prior therapies achieved PR. In 2006, FDA approved vorinostat for the treatment of CTCL patients who have progressive, persistent or recurrent disease on or following two systemic therapies. Also in Japan, the drug was approved in 2011 based on the phase 1 clinical trial conducted in Japan (58). In a recent phase 3 randomized study, vorinostat was compared with mogamulizumab in patients with stage IB-IV MF/SS (8). The ORR for the vorinostat was significantly lower than that of mogamulizumab (5 vs. 28%).

Romidepsin is a bicyclic peptide that inhibits class I HDAC selectively. Preclinical studies suggest that romidepsin is among the most potent HDAC inhibitors. Two multicenter phase 2 clinical trials of romidepsin for CTCL were conducted before 2010. In one clinical trial, 71 refractory IA-IVB MF/SS patients with a median of four prior treatments were enrolled (19). Some patients received 18 mg/m^2^ romidepsin on days 1 and 5 of a 21-day cycle and to other patients romidepsin was administered at 14 mg/m^2^ on days 1, 8, and 15 every 28 days. CR was observed in four patients (5.6%) and 20 patients achieved PR (28.2%). The median DOR was 13.7 months. In the other international single-arm, open-label, phase 2 study, 96 patients with IB-IVA MF/SS who had received one or more prior systemic therapies (median three), received romidepsin intravenously 14 mg/m^2^ on days 1, 8, and 15 every 28 days (20). The ORR was 34% (33/96), including 6% (6/96) CRs and the median DOR was 15.0 months, which were similar to the previous study. Interestingly, in the clinical trial, romidepsin is active in subtypes of CTCL with less favorable outcomes, such as tumor stage and folliculotropic MF. The ORR was 45% (9/20) in patients with cutaneous tumors and 60% (6/10) in patients with folliculotropic disease involvement (59). Of note, Kim et al. reported that a clinically significant effect on pruritus was confirmed in a large number of patients, even in patients without any objective clinical response (60). In 2009, romidepsin was approved for the treatment of CTCL patients by FDA.

Panobinostat is an orally bioavailable pan HDAC inhibitor approved for the treatment of multiple myeloma by FDA in 2015. In a phase 2 study, 139 patients with stage IB-IVA MF/SS who had been pretreated with two or more prior systemic therapies, received 20 mg of oral panobinostat three times every week (21). The 139 patients included 79 bexarotene-exposed patients and 60 bexarotene-naïve patients. The ORR was 17.3% in all patients (15.2% in the bexarotene-exposed group and 20.0% in the bexarotene-naïve group). One CR was observed in each group. The median PFS was 4.2 months in the bexarotene-exposed group and 3.7 months in the bexarotene-naïve group. The median DOR was 5.6 months in the bexarotene-exposed group and was not reached at data cutoff in the bexarotene-naïve group.

Belinostat is an intravenous inhibitor of pan HDAC, which was approved for the treatment of relapsed or refractory PTCL by FDA in 2014. In the phase 2 clinical trial of belinostat in patients with relapsed or refractory PTCL and CTCL, 29 patients with CTCL including 17 MF patients and seven SS patients were enrolled. Patients with CTCL had received a median of four prior systemic therapies. Belinostat was administered at 1,000 mg/m^2^ intravenously for consecutive 5 days of a 21-day cycle (22). The ORR was 13.8% (10.3% CR and 3.4% PR), and the median DOR was 83 days.

Resminostat is an oral drug which selectively inhibits class I, IIB, and IV HDAC enzymes. A phase 2, multicenter, double-blind, randomized, placebo-controlled trial is currently ongoing to evaluate whether resminostat can be used as maintenance treatment for MF/SS patients after disease control with other systemic therapies (NCT02953301). Patients will receive either placebo or 600 mg resminostat for consequent 5 days followed by 9 days of rest in a 14-day cycle. This clinical trial will be completed in 2020.

## Pralatrexate

Pralatrexate, an anti-neoplastic folate analog, inhibits dihydrofolate reductase, targeting DNA synthesis and resulting in tumor cell death. Pralatrexate has the improved anti-tumor activity compared to methotrexate due to higher affinity for the reduced folate carrier-1 and more selective accumulation in tumor cells.

A phase 2 study of pralatrexate in 109 patients with PTCL including 12 transformed MF patients who progressed following one or more prior systemic therapy (PROPEL study) showed that the ORR was 29% (32 of 109), including 11% CR and 18% PR, with the median DOR of 10.1 months. The median PFS and overall survival (OS) were 3.5 and 14.5 months, respectively (61). Subgroup analysis patients with transformed MF revealed that the ORR was 58% with the median DOR and PFS were 4.4 and 5.3 months, respectively per investigator assessment (24). Pralatrexate was administered at 30 mg/m^2^/week for 6 weeks followed by one week of rest (7-week cycle) in this study. FDA approved pralatrexate for the treatment of PTCL in 2009. In Japan, after phase 1/2 clinical study was conducted, pralatrexate was approved in 2018 (62).

As for CTCL, a dose de-escalation study of pralatrexate showed that the recommended regimen was identified as 15 mg/m^2^/week for 3 weeks followed by 1 week of rest (4-week cycle) (63). Twenty-nine patients with refractory MF/SS and primary cutaneous ALCL with at least one prior systemic therapy received recommended dosing regimen. The ORR was 45% with 1 CR and 12 PR. In any study, the most observed toxicity is mucositis. To reduce this risk, patients received supplementation of vitamin B12 and folate, and leucovorin (folinic acid) during pralatrexate treatment. Pralatrexate can be a promising treatment with the potential to provide lasting benefit for advanced CTCL patients with the relative low toxicity. Recently, a phase 1/2 study suggested that combination therapy of 150 mg/m^2^ daily bexarotene plus 15 mg/m^2^/week for 3/4 weeks pralatrexate is active with high ORR (60%) and minimal toxicity for CTCL (64). A phase 1 study of pralatrexate (10 to 25 mg/m^2^) and romidepsin (12 to 14 mg/m^2^) on 1 of 3 schedules: every week × 3 every 28 days, every week × 2 every 21 days, and every other week every 28 days, for patients with PTCL also showed high ORR (57%) (65). These combination therapies with pralatrexate plus bexarotene or romidepsin can be an efficient and tolerated treatment option.

## Forodesine

Purine nucleoside phosphorylase (PNP) is an important enzyme for the phosphorolysis of purine nucleosides. Severe immunodeficiency syndromes are caused by congenital defects in this enzyme through selective depletion of T cells but not of B cells (66, 67). Based on increased nucleoside metabolism of malignant T cells, T-cell tumor cells can be highly sensitive to the inhibition of PNP (68). Forodesine is a potent inhibitor of PNP that causes apoptosis in both neoplastic T cells and normal T cells.

In a multicenter phase 2 open-label study, 144 patients with MF/SS who had been treated with three or more systemic therapies were enrolled. The patients received oral forodesine 200 mg daily. The drug showed limited clinical activity in this study. No CRs were observed, and only 11% of the patients achieved PR and 50% maintained SD. The median DOR was 191 days (25). Although almost all patients (96%) experienced at least one AE, most AEs were grade 1/2. Common AEs were peripheral edema, fatigue, insomnia, pruritus, diarrhea, headache, and nausea.

Forodesine was approved in Japan for the treatment of PTCL at the dose of 600 mg daily based on efficacy and safety results of the phase 1/2 clinical trial in patients with 48 relapsed PTCL including one transformed MF patient (65). In 41 evaluable patients, the ORR was 25% including 4 CRs. The most common grade 3/4 AEs were lymphopenia (96%), leukopenia (42%), and neutropenia (35%). Dose reduction and discontinuation due to AEs were uncommon. There is a possibility that such high-dose can be an effective and acceptable treatment for advanced MF/SS.

## Denileukin Diftitox

Denileukin diftitox is a genetically engineered fusion protein combining the full-length sequence of human IL-2 with the cytotoxic and membrane-translocating domains of the diphtheria toxin. After binding to the IL-2 receptor (IL-2R) on neoplastic T cells, the drug is internalized. The diphtheria toxin results in the production of a single polypeptide chain that is capable of inhibiting protein synthesis in the cells, leading to cell death (69). The human IL-2R consisted of three forms: low, intermediate, and high affinity. The high affinity IL-2R is a complex of distinct proteins of α chain (CD25), β chain (CD122), and γ chain (CD132). The intermediate one is composed of CD122 and CD132, and CD25 alone defines the low affinity one. Although denileukin diftitox can bind to all forms of the IL-2R, internalization is caused by only intermediate or high affinity receptors (70). In addition, it is known that the baseline expression level of CD25, which is not included in the intermediate affinity IL-2R, on CTCL cell in lesional skin correlated with their clinical response to denileukin diftitox (71), suggesting that the high affinity IL-2R is the most important receptor to elicit an effect.

The largest study of denileukin diftitox was a multicenter, randomized, double-blind placebo-controlled phase 3 trial that evaluated denileukin diftitox (9 or 18 μg/kg/day) vs. placebo in 144 stage IA-III MF/SS patients who had been treated with at most three prior therapies (26). The trial excluded patients with low CD25 expression disease (defined as detectable CD25 on <20% of T cells in lesional skin). The drugs were administered for consequent 5 days every 3 weeks for up to eight cycles. The ORR for the denileukin diftitox 18 μg/kg/day group was 49.1% with 9.1% CR (*n* = 55), compared with 15.9% with 2.3% CR for placebo (*n* = 44). For the denileukin diftitox 9 μg/kg/day group, the ORR was 37.8% (*n* = 45; 11.1% CR and 26.7% PR). The PFS was significantly prolonged for denileukin diftitox-treated patients compared to patients treated with placebo. Estimated median PFS was at least 971 days for the denileukin diftitox 18 μg/kg/day cohort, 794 days for the denileukin diftitox 9 μg/kg/day cohort, and only 124 days for placebo cohort. The drug-related severe AEs occurred in 25% of the participants receiving denileukin diftitox with premedication of acetaminophen and antihistamine. The most common drug-related severe AEs were dehydration (2%) and capillary leak syndrome (2%). To assess the denileukin diftitox effect on patients with low CD25 expression, 36 patients with MF/SS who had been excluded from the placebo-controlled trial due to low CD25 expression were enrolled in another clinical trial. In the clinical trial, patients were treated with denileukin diftitox 18 μg/kg/day for 5 consecutive days every 3 weeks for up to eight courses. The ORR was 30.6% (8.3% CR and 22.2% PR) (72). This study suggests that low CD25 expression does not necessarily preclude a meaningful clinical response to denileukin diftitox in patients with CTCL.

Denileukin diftitox had been approved by the FDA in 1999 for the treatment of patients with CTCL refractory to standard treatment options. However, denileukin diftitox is unavailable on the global market at this time. Currently, the related agent E7777 which shares an amino acid sequence with denileukin diftitox but has improved its purity and an increased percentage of active protein monomer species is being evaluated. A phase 1 study for 13 patients with PTCL conducted in Japan showed that E7777 is well-tolerated and has antitumor activity with 38% ORR (73). A phase 2 clinical trial of E7777 for relapsed or refractory PTCL and CTCL (NCT02676778) and a phase 3 clinical trial for persistent and recurrent CTCL (NCT01871727) are ongoing.

## Duvelisib

Phosphoinositide-3-kinase (PI3K) is a lipid kinase involved in intracellular signal transduction and regulates multiple cellular functions relevant to oncogenesis. The PI3K-δ and PI3K-γ isoforms, which are preferentially expressed in leukocytes, can modulate both innate and adaptive immune response (74–77). PI3K-δ and PI3K-γ mediate multiple pathways contributing to survival, proliferation, and differentiation in malignant hematopoietic cells. Moreover, PI3K signaling is involved in development of tumor microenvironment through juxta-, para-, and endocrine effects on stromal and immune cells (78–80). Additionally, PI3K-γ may also suppress antitumor immune response by inhibiting phagocytosis by tumor-associated macrophages (81). Thus, there are at least three different mechanisms via which PI3K-δ and PI3K-γ inhibitors could be effective for hematopoietic malignancies.

Duvelisib (also known as IPI-145) is an oral, dual inhibitor of PI3K-δ and PI3K-γ. In a recent phase 1 open-label trial, clinical activity of duvelisib was promising and the toxicity was acceptable in relapsed or refractory PTCL and CTCL (27). Thirty-five patients (16 PTCL, 19 CTCL) were enrolled in this study and 27 (77%) were treated at the maximum tolerated of oral duvelisib 75 mg twice daily on a 28-day cycle. The 19 patients with CTCL had received a median of six prior therapies. The CTCL population was composed of 13 patients with MF, five patients with SS, and one primary cutaneous ALCL patient. In the CTCL population, the ORR was 31.6% (all PR) and the median PFS was 4.5 months. The most common grade 3/4 AEs were increase of liver enzymes (40%), neutropenia (17%), maculopapular rash (17%), and pneumonia (17%). Thus, this study suggests that duvelisib has clinical activity with an acceptable toxicity, while further studies are needed to determine the optimal dose and identify an appropriate combination therapy. A phase 1/1b clinical trial of the other dual inhibitor of PI3K-δ and PI3K-γ, RP6530, in relapsed and refractory T-cell Lymphoma has been finished, but data analysis is incomplete (NCT02567656).

## Lenalidomide

Lenalidomide, a derivative of thalidomide, is an oral immunomodulatory drug with direct immune-mediated mechanism (82). Lenalidomide has been shown to induce growth arrest and apoptosis in lymphoma cell lines and FDA approved the drug for the treatment of myelodysplastic syndrome, refractory/relapsed multiple myeloma, and mantle cell lymphoma (83, 84). In addition, lenalidomide is currently being used in clinical trials to treat other various hematopoietic malignancies.

A multicenter phase 2 study of lenalidomide in 32 patients with MF/SS who progressed following a median of 4 systemic therapies was conducted between 2005 and 2010 (28). The first 19 patients received lenalidomide at a daily dose of 25 mg orally for 21 days of a 28-day cycle. The remaining 13 patients initiated treatment at a dose of 10 mg daily and the dose was then increased by 5 mg every 28 days to a maximum of 25 mg daily, based on patient safety and response. The ORR was 28% (all PR) with the median PFS of 8 months. The most frequent AEs were lower leg edema, anemia, fatigue, and transient flare reaction that mimic worsening of the patient's disease. Patients with a 25-mg starting dose showed AEs more frequently than those with a 10-mg starting dose. In a phase 3 randomized study of lenalidomide maintenance vs. observation alone after disease control with other therapies in 21 advanced CTCL patients, the median PFS was 5.3 months in the maintenance lenalidomide group (*n* = 9) and 2 months in the observation alone group (*n* = 12) (85). Because lenalidomide was used as a maintenance therapy, ORR was not evaluated. The main AEs noted in the lenalidomide arm were neutropenia, erythema multiforme, periorbital edema, hypothyroidism, and pruritus. Although statistical comparison in this study was severely underpowered, lenalidomide may be used as a maintenance therapy after debulking therapy.

## Everolimus

Everolimus is an oral agent that targets the mammalian target of rapamycin (mTOR) pathway. The mTOR regulates several survival and growth pathways in a variety of cancers, which was also shown for T-cell non-Hodgkin lymphoma. In addition, an immunohistochemical study revealed that activation of mTOR pathway in MF is associated with the acquisition of a more aggressive phenotype (86). In the recent phase 2 clinical trial, 16 patients with relapsed or refractory T-cell lymphoma including 7 patients with MF were enrolled and received oral everolimus 10 mg daily. The ORR was 44% and the median PFS was 4.1 months (29). Regarding MF, three of seven patients showed PR and none reached CR. The most frequent AEs were hematologic toxicity and skin rash.

## Conclusion

**Figure 1:**
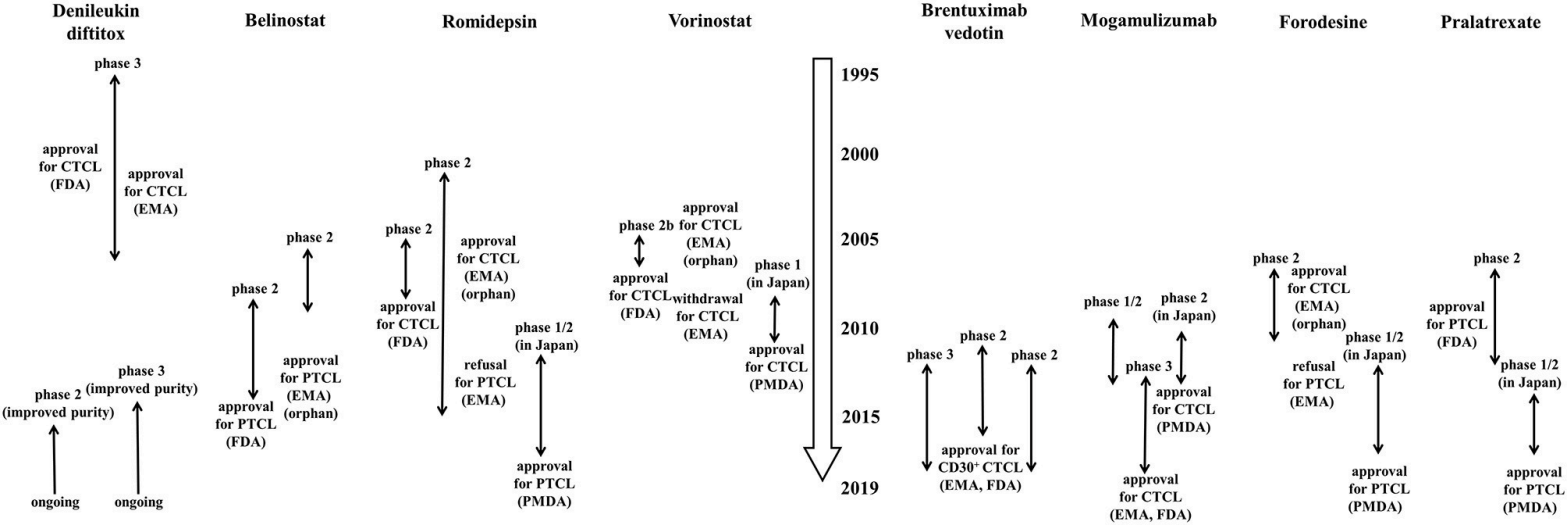
History of clinical trials of single-agents which have been approved for cutaneous T-cell lymphoma or peripheral T-cell lymphoma by FDA, EMA, or PMDA. The data were collected on March 31, 2019. When the drug was approved as orphan drug from EMA, the comment “orphan” is added. CTCL, cutaneous T-cell lymphoma; PTCL, peripheral T-cell lymphoma; FDA, food and drug administration; EMA, European medicines agency; PMDA, pharmaceuticals and medical devices agency.

**Figure 2:**
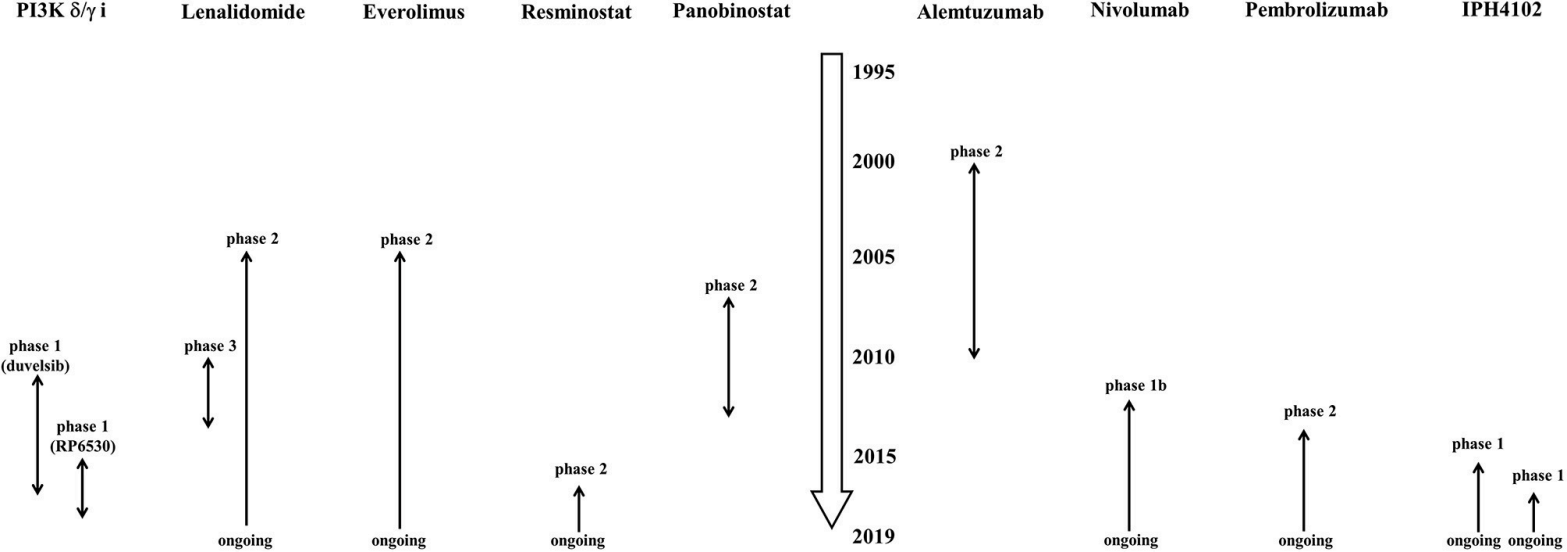
History of clinical trials of single-agents which have not been approved for cutaneous T-cell lymphoma or peripheral T-cell lymphoma by FDA, EMA, or PMDA. The data were collected on March 31, 2019. PI3K δ/γ I, phosphoinositide-3-kinase δ/γ inhibitor.

Although many patients with early CTCL have slow-progressing disease with a normal life expectancy, prognosis of patients with advanced stages of CTCL is poor. Generally speaking, CTCL is incurable without allogeneic stem cell transplantation. Current treatment outcome is characterized by high relapse rates and low durable remission rates. As treatment of advanced-stage CTCL is mostly palliative and not curable, a stage-based approach utilizing sequential therapies in an escalated manner is currently favorable. Existing clinical practice guidelines are quite heterogeneous. Consequently, therapeutic decisions should be individualized to each patient by means of a risk-proportionate approach. Although many novel therapeutic agents have been developed and clinical trials for CTCL and PTCL had or have been implemented (Figures 1, 2), such drugs also showed limited efficacy as reviewed in this paper. Thus, it is necessary to know which therapy is preferable for each patient with MF/SS. Creatively designed international clinical trials, such as MAVORIC study and ALCANZA study, should be encouraged.

## Author Contributions

TO conceived the concept and wrote the manuscript. TM co-conceived the concept, edited and improved the manuscript, and drafted the table.

### Conflict of Interest Statement

The authors declare that the research was conducted in the absence of any commercial or financial relationships that could be construed as a potential conflict of interest.

## Abbreviations

CTCL: cutaneous T-cell lymphoma
PTCL: peripheral T-cell lymphoma
MF: mycosis fungoides
SS: Sézary syndrome
ALCL: anaplastic large cell lymphoma
AE: adverse event
ORR: overall response rate
CR: complete response
PR: partial response
SD: stable disease
PFS: progression free survival
OS: overall survival
DOR: duration of response
CCR4: C-C chemokine receptor 4
HDAC: histone deacetylase
PNP: purine nucleoside phosphorylase
MMAE: monomethyl auristatin E
IL-2R: IL-2 receptor
PI3K: Phosphoinositide-3-kinase
FDA: Food and Drug Administration
EMA: European Medicines Agency
CTLA-4: cytotoxic T lymphocyte-associated protein 4
PD-1: programmed cell death protein 1
mTOR: mammalian target of rapamycin.
